# Exploring the association between thyroid- stimulating hormone and metabolic syndrome: A large population-based study

**DOI:** 10.1371/journal.pone.0199209

**Published:** 2018-06-21

**Authors:** Yi-Chao Zhou, Wen-Hui Fang, Tung-Wei Kao, Chung-Ching Wang, Yaw-Wen Chang, Tao-Chun Peng, Chen-Jung Wu, Hui-Fang Yang, James Yi-Hsin Chan, Wei-Liang Chen

**Affiliations:** 1 Division of Family Medicine, Department of Family and Community Medicine, Tri-Service General Hospital; and School of Medicine, National Defense Medical Center, Taipei, Taiwan, Republic of China; 2 Health Management Center, Department of Family and Community Medicine, Tri-Service General Hospital, National Defense Medical Center, Taipei, Taiwan, Republic of China; 3 Division of Geriatric Medicine, Department of Family and Community Medicine, Tri-Service General Hospital; and School of Medicine, National Defense Medical Center, Taipei, Taiwan, Republic of China; 4 Division of Family Medicine, Department of Community Medicine, Taoyuan Armed Forces General Hospital, Taoyuan, Taiwan, Republic of China; 5 Graduate Institute of Medical Sciences, National Defense Medical Center, Taipei City, Taiwan, Republic of China; The University of Tokyo, JAPAN

## Abstract

A growing amount of evidence suggests that thyroid-stimulating hormone (TSH) is associated with cardiometabolic risk. However, there have been few longitudinal studies. The aim of this study was to explore the causal relationship between TSH and metabolic syndrome (MetS) in a large population-based longitudinal study. From 2010 to 2016 at the Health Management Center at Tri-Service General Hospital, 25,121 eligible patients were enrolled in our cross-sectional analyses. Cox proportional hazard models were used to investigate the longitudinal association among hypertension (HTN), prediabetes (pre-DM), MetS, diabetes (DM) and TSH levels (N = 12,463). The average follow-up time was 7.2 years. In the cross-sectional analysis, the OR for MetS was 1.06 (95% CI = 1.03–1.09; P< 0.05), while the ORs for DM, pre-DM or HTN were not statistically significant (all P> 0.05). After dividing TSH levels into four quartiles, the ORs for the presence of MetS determined by comparing the highest TSH quartile with the lowest TSH quartile were 1.37 (95% CI = 1.18–1.60), 1.42 (95% CI = 1.20–1.67), and 1.44 (95% CI = 1.22–1.69) (all, P<0.05) in model 1, model 2 and model 3 respectively. The HR for the incidence of MetS was 1.33 (95% CI = 1.17–1.51; P < 0.05). Our study revealed that TSH levels had a strong association with incident MetS.

## Introduction

MetS is a cluster of metabolic abnormalities including central obesity, hypertension, dyslipidemia and hyperglycemia [1]. The presence of MetS is closely related to a substantially increased risk of developing serious diseases such as type 2 diabetes mellitus and cardiovascular disease [2]. Recently, an increasing prevalence of MetS has been reported worldwide, including in Asian countries and in Taiwan [3–5]. Accordingly, predicting the occurrence of MetS is crucial for the prevention of subsequent irreversible metabolic diseases. Notably, higher TSH levels have been reported to be associated with the presence of obesity, increased triglycerides and a higher risk of MetS [6, 7]. Positive associations between TSH levels and systolic blood pressure(SBP) and diastolic blood pressure(DBP) were also found in a population-based study [8]. Numerous studies have shown increased TSH levels are associated with less favorable lipid profiles in euthyroid subjects [9–11]. Recent studies have revealed that slight elevations in TSH levels in patients with subclinical hypothyroidism, or high normal TSH levels in euthyroid patients are associated with higher risk of developing MetS [6]. In contrast, some reports have shown that there is no association between TSH and MetS in euthyroid subjects and no associations between peripheral thyroid hormones and MetS [7, 12, 13].

To the best of our knowledge, it is not well -understood whether a higher TSH level within the euthyroid range or subclinical hypothyroidism is associated with an increased prevalence and incidence of MetS. Comparisons of four other longitudinal studies that attempted to explore the association between TSH and MetS in euthyroid populations were performed in the present study. Two studies involved Korean populations [14, 15], and the other two studies were performed in Iran and in the USA population [16, 17]. There were strengths and weaknesses of those four studies. Consequently, the purpose of this study was to further clarify the association between TSH levels and the presence of MetS by conducting cross-sectional and longitudinal cohort analyses.

## Methods

### Study subjects

**Fig 1:**
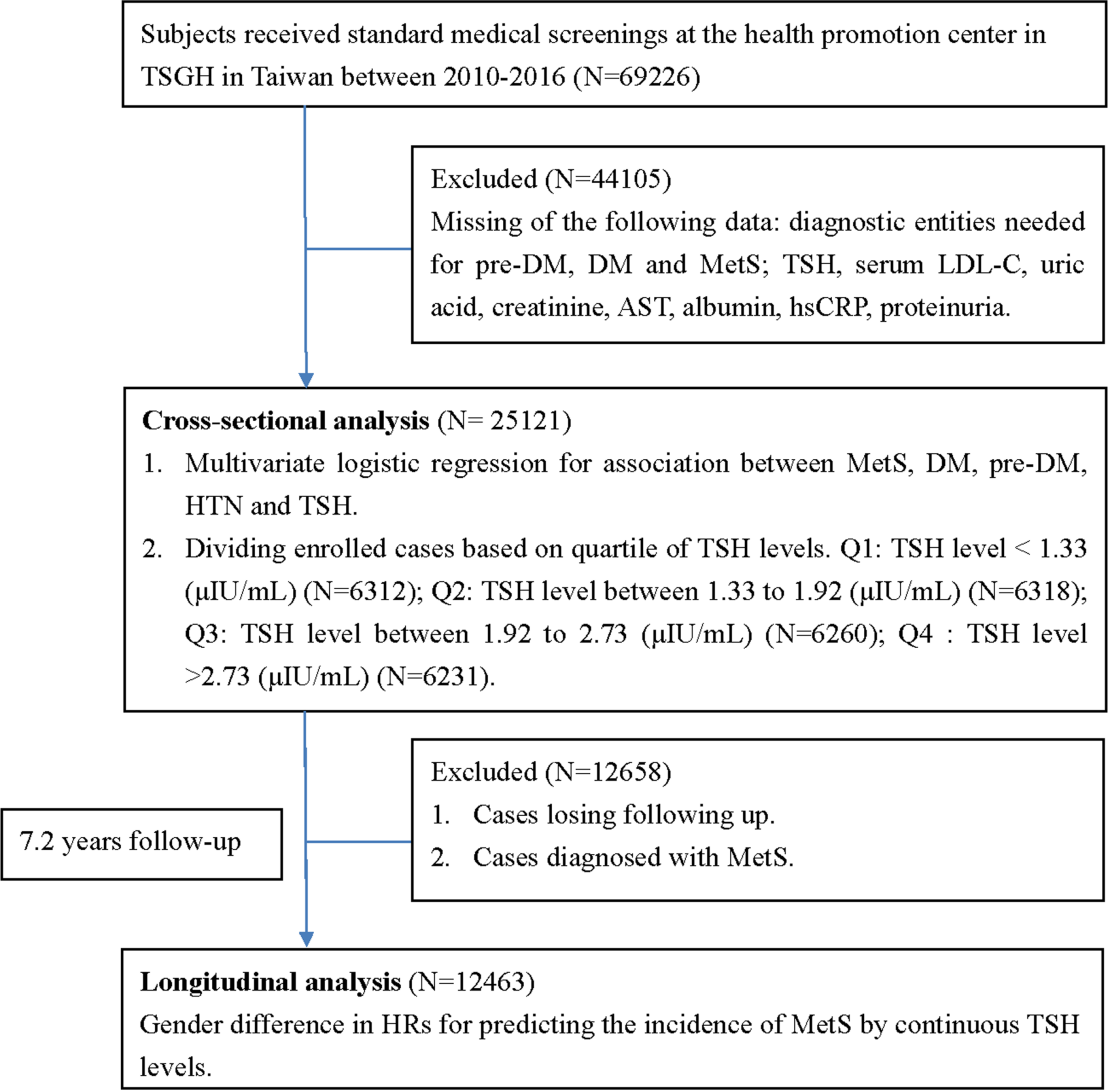
Flow diagram of our study.

Our study data were extracted from 69,226 participants who underwent health examinations at the Health Management Center in Tri-Service General Hospital (TSGH), Taiwan, from 2010 to 2016. This study was approved by the institutional review board (IRB) of TSGH and was performed in accordance with the revised Helsinki Declaration. The IRB waived the need to obtain individual informed consent because the data were analyzed anonymously. Participants with missing data including TSH levels, serum LDL-C, uric acid, creatinine, AST, albumin, hsCRP, proteinuria, and diagnostic tests for prediabetes, DM, and MetS were all excluded (N = 44,105). The final sample contained 25,121 eligible subjects for the cross-sectional analysis. We also excluded patients (N = 12,658) who were diagnosed with MetS or who were lost to follow-up for further analyses. The final sample contained 12,463 eligible subjects in a longitudinal analysis. Our study design and the selection of participants are shown in Fig 1.

### TSH measurement

TSH levels were measured acquired by Cisbio Bioassays with an accurate immunoradiometric assay that is based on the sandwich type principle.

### Definition of HTN

Blood pressure was measured by a sphygmomanometer. A blood pressure of 140/90 mmHg or the use of medication for previously diagnosed hypertension was defined as hypertension.

### Definition of prediabetes

Prediabetes was diagnosed according to the American Diabetes Association criteria as long as one of the following criteria was met [18]: a fasting plasma glucose between 100 mg/dl and 125 mg/dl or an HbA1c level between 5.7% to 6.4%.

### Definition of Type 2 DM

Type 2 DM was defined according to the American Diabetes Association criteria as long as one of the following criteria was met [19]: hemoglobin A1c test ≥ 6.5%; fasting plasma glucose ≥ 126 mg/dL; 2-hour plasma glucose ≥200 mg/dl; classic symptoms of hyperglycemia or a hyperglycemic crisis; or a random serum glucose ≥200 mg/dl. Participants with a past history of diabetes status and those who used antidiabetic agents were also considered to have DM.

### Criteria for MetS

According to the criteria of the International Diabetes Federation [20], participants were diagnosed with MetS if they had central obesity based on waist circumference (>90 cm in men and >80 cm in women in Taiwan) [21] and met two or more of the following diagnostic criteria: (1) triglycerides (TG) ≥150 mg/dL; (2) high-density lipoprotein cholesterol (HDL-C) <40 mg/dL in men and <50 mg/dL in women; (3) SBP ≥130 mmHg or DBP ≥85 mmHg, or the current use of any antihypertensive medications; or (4) a fasting serum glucose ≥100 mg/dL or a previous diagnosis of T2DM, or the current use of any antidiabetic medications.

### Covariate measurements

The health examinations included standard evaluations of physical and mental health, comprehensive biochemistry tests, and anthropometric measurements. Biochemical parameters affecting metabolic values were corrected by having the patients fast for at least 8 hours. Age, sex, smoking history, exercise habits, drinking history and medical history were self-reported by the participants. Drinking alcohol was defined through self-report questionnaire and was categorized as ‘‘never” and ‘‘alcohol consumption”. Exercise habits were classified according to the question “How often do you exercise?”. “Exercise” was defined in subjects who self-reported to exercise at least 1–2 times every week. Body weight and height were obtained from a digital scale. Body mass index (BMI) was calculated by dividing the body weight with the square of the body height (kg/m2). The presence of proteinuria was obtained with a urine dipstick colorimetric test. Other biochemical parameters, including serum glucose, serum TG, serum low-density lipoprotein cholesterol (LDL-C), serum HDL-C, serum creatinine, high sensitivity CRP (hsCRP), serum uric acid, serum albumin and aspartate aminotransferase (AST) were measured by the HK UV test, the GPO-POD -method, the liquid selective detergent method, the accelerator selective detergent method, a kinetic-modified Jaffe method using alkaline picrate, a near- infrared particle immunoassay rate method, the uricase PAP method, the bromocresol green method and the kinetic UV method (Tris buffer without P5P), respectively. Body composition exams performed during the health evaluations included measurements of the percentage of percentage of body fat. BIA (InBody720, Biospace, Inc., Cerritos, CA, USA), which is a commonly used and effective method for assessing body composition, was performed in this study.

### Statistical analysis

All statistical analyses were performed with the Statistical Package for the Social Sciences, version 22.0 for Windows. The mean and standard deviation (SD) were calculated for continuous variables, whereas frequencies and percentages were calculated for discrete variables. Student's *t*-test and the Chi-square test were utilized for discrete data and continuous data, respectively. A two-sided P-value less than 0.05 was considered statistically significant. We applied a quartile-based analysis in our study by stratifying TSH levels into four quartiles. The cut-off levels for TSH were as follows: Q1 < 1.33 (μIU/mL), 1.33 ≤ Q2 < 1.92 (μIU/mL), 1.92 ≤ Q3 < 2.73 (μIU/mL), and 2.73 ≤ Q4 (μIU/mL). Multivariate logistic regression was performed to evaluate the associations between TSH levels and the prevalence of HTN, prediabetes, MetS and DM. Covariate adjustment was used, and the extended statistical results were evaluated with the following three models: model 1 = unadjusted; model 2 = adjusted by age, gender, proteinuria, LDL-C, uric acid, creatinine, AST, albumin, and hsCRP; model 3 = model 2 + adjustment for smoking and drinking history. A Cox proportional hazard model was generated to evaluate the association between continuous TSH levels and the incidence of MetS.

## Results

The demographic characteristics of the 25,121 enrolled participants stratified by TSH quartiles are listed in Table 1. Patients with Q1TSH were more likely to be younger and male, have a history of smoking and have proteinuria, lower LDL-C, and lower AST than patients with Q4 TSH (all, P<0.05). Table 2 showed the ORs for the presence of HTN, prediabetes, MetS and DM as estimated by TSH levels; the ORs for MetS in all model 1, model 2 and model 3 were 1.06 (95% CI: 1.03 to 1.09), 1.05 (95% CI: 1.02 to 1.09), 1.06 (95% CI: 1.06 to 1.09), respectively (all P < 0.05), while the ORs for HTN, prediabetes, MetS and DM in all models were not statistically significant (all P > 0.05). These findings illustrated that MetS had a stronger association with TSH levels than HTN, prediabetes and DM. After stratification of TSH levels into four quartiles, the estimated presence of MetS in patients with Q4TSH was significantly different with Q1 TSH. (Table 3, all P<0.0001). Table 4 showed the HRs for the presence of MetS in model 1, model 2 and model 3 were 1.33 (95% CI: 1.17 to 1.51), 1.21 (95% CI: 1.04 to 1.40), 1.18 (95% CI: 1.02–1.37) respectively (all P < 0.05). Furthermore, the HRs for incident MetS in females were 2.26 (95% CI: 1.53 to 3.34), 2.49 (95% CI: 1.40 to 4.41), 2.52 (95% CI: 1.35–4.68) in model 1, model 2 and model 3, respectively (all P < 0.05). These findings conclusively suggested that the TSH level might be a useful tool for predicting the incidence of MetS in females.

**Table 1:** Demographic and characteristics of enrolled participants and their association between TSH levels which were divided into four groups.

Variables	Q1 TSH (n = 6312)	Q2 TSH (n = 6318)	Q3 TSH (n = 6260)	Q4 TSH (n = 6231)
**Continuous Variables, mean (SD)**				
** Age**	48.55(15.15)	48.13(14.69)	48.68(15.08)	50.66(15.69)*
** LDL-C**	185.31(35.40)	186.69(35.20)	188.29(36.16)*	190.04(36.84)*
** Uric acid**	5.71(1.46)	5.74(1.46)	5.74(1.47)	5.71(1.52)
** Creatinine**	0.84(0.29)	0.85(0.24)	0.85(0.30)	0.87(0.48)*
** AST**	20.97(11.95)	21.48(14.04)	21.66(11.57)*	21.85(10.85)*
** Albumin**	4.45(0.28)	4.47(0.28)*	4.46(0.29)	4.45(0.30)
** hsCRP**	0.24(0.61)	0.23(0.45)	0.24(0.51)	0.23(0.42)
** Fat percentage**	27.42(7.31)	27.56(7.21)	28.06(7.22)*	29.00(7.63)*
**Category Variables, (%)**				
** Proteinuria**	1594(27.9)	1480(26.1)	1443(25.9)	1392(25.0)
** Male**	3590(60.9)	3538(60.6)	3267(57.0)	2845(50.4)
** Smoking**	1504(34.0)	1351(29.9)	1274(28.3)	1152(25.5)
** Drinking**	1194(48.0)	2016(48.7)	1937(46.9)	1906(46.5)
** Exercise**	1965 (31.1)	2009 (31.8)	1965 (31.4)	1904 (30.6)

Abbreviation: SD, standard deviation; LDL-C, low density lipoprotein cholesterol; hsCRP, high sensitivity C-reactive protein; AST, aspartate aminotransferase.

* indicates TSH quartiles (Q2, Q3, Q4) with different letters were significantly different from Q1 TSH (p < 0.05, ANOVA).

**Table 2:** Association between TSH (as continuous variables) and MetS, DM, pre-DM, HTN.

Variable	Model 1		Model 2		Model 3	
	OR (95% CI)	*P* value	OR (95% CI)	*P* value	OR (95% CI)	*P* value
**TSH**	**HTN**					
	1.00 (0.97–1.04)	0.875	0.99 (0.96–1.03)	0.659	0.99 (0.96–1.03)	0.583
	**Prediabetes**					
	1.02 (0.99–1.05)	0.202	1.01 (0.98–1.05)	0.564	1.01 (0.98–1.05)	0.586
	**DM**					
	1.03 (0.98–1.08)	0.259	1.02 (0.97–1.08)	0.413	1.02 (0.97–1.08)	0.433
	**MetS**					
	1.05 (1.02–1.08)	0.001	1.05 (1.01–1.09)	0.007	1.05 (1.01–1.09)	0.009

Model 1: unadjusted.

Model 2: adjusted by (age, gender, BMI, proteinuria, LDL-C, serum uric acid, serum creatinine, serum AST, serum albumin, hsCRP, fat percentage, excercise).

Model 3: adjusted by Model 2+ (smoking, drinking)

**Table 3:** Comparing higher TSH quartiles with the lowest TSH quartile to explore their association with HTN, prediabetes, MetS, and DM.

TSH Quatiles	Model 1		Model 2		Model 3	
	ORs (95% CI)	*P*	ORs (95% CI)	*P*	ORs (95% CI)	*P*
**HTN**						
Q2 vs Q1	1.04 (0.87–1.23)	0.682	1.02 (0.85–1.22)	0.860	1.01 (0.84–1.21)	0.931
Q3 vs Q1	1.01 (0.85–1.20)	0.914	1.01 (0.84–1.21)	0.917	1.00 (0.83–1.20)	0.995
Q4 vs Q1	1.08 (0.92–1.28)	0.353	1.02 (0.85–1.22)	0.858	1.00 (0.83–1.20)	0.980
**Prediabetes**						
Q2 vs Q1	0.97 (0.81–1.17)	0.776	0.95 (0.78–1.15)	0.608	0.96 (0.79–1.16)	0.640
Q3 vs Q1	1.04 (0.86–1.24)	0.706	1.03 (0.85–1.24)	0.792	1.03 (0.85–1.25)	0.751
Q4 vs Q1	0.97 (0.81–1.17)	0.770	0.89 (0.73–1.08)	0.226	0.89 (0.73–1.08)	0.224
**DM**						
Q2 vs Q1	1.15 (0.86–1.53)	0.360	1.16 (0.85–1.57)	0.345	1.19 (0.88–1.61)	0.271
Q3 vs Q1	1.00 (0.74–1.35)	0.986	1.02 (0.74–1.39)	0.915	1.03 (0.75–1.41)	0.847
Q4 vs Q1	1.19 (0.89–1.59)	0.238	1.11 (0.82–1.50)	0.515	1.12 (0.82–1.52)	0.474
**MetS**						
Q2 vs Q1	1.10 (0.93–1.30)	0.271	1.08 (0.90–1.31)	0.413	1.11 (0.91–1.34)	0.307
Q3 vs Q1	1.18 (1.01–1.40)	0.044	1.22 (1.01–1.48)	0.041	1.24 (1.03–1.51)	0.025
Q4 vs Q1	1.40 (1.19–1.65)	<0.0001	1.34 (1.11–1.61)	0.003	1.35 (1.12–1.64)	0.002

Model 1: unadjusted.

Model 2: adjusted by (age, gender, BMI, proteinuria, LDL-C, serum uric acid, serum creatinine, serum AST, serum albumin, hsCRP, fat percentage, exercise).

Model 3: adjusted by Model 2+ (smoking, drinking)

**Table 4:** Gender differences in HRs of MetS by TSH quartiles.

		Model 1		Model 2		Model 3	
		HR (95% CI)	P Value	HR (95% CI)	P Value	HR (95% CI)	P Value
MetS	Total	1.31 (1.16–1.49)	<0.0001	1.19 (1.02–1.38)	0.023	1.17 (1.00–1.37)	0.044
	Male	1.21 (1.01–1.44)	0.038	1.13 (0.93–1.36)	0.211	1.12 (0.93–1.36)	0.226
	Female	2.36 (1.54–3.61)	<0.0001	2.47 (1.33–4.59)	0.004	2.70 (1.34–5.43)	0.005

Model 1: unadjusted.

Model 2: adjusted by (age, BMI, proteinuria, LDL-C, serum uric acid, serum creatinine, serum AST, serum albumin, hsCRP, fat percentage, exercise)

Model 3: adjusted by Model 2+ (smoking, drinking)

## Discussion

There were several remarkable findings in the present large-scale population-based cohort study. First, continuous TSH levels had a significant association with the presence of MetS. This association grew stronger when the highest TSH quartile was compared with the lowest TSH quartile. Notably, after a 7.2-year follow-up period, the continuous TSH level was a significant risk factor for developing MetS. To the best of our knowledge, this is the first study in Asian population to address the utilization of the TSH level as a predictive tool for both prevalent and incident MetS.

After reviewing the published articles, four other longitudinal studies exploring the association between MetS and thyroid function were identified (Table 5). The only one with time-to-event analysis as in the present study was performed by Mehran L et al[16]. Their study contained 2,393 participants and revealed that a decrease in FT4 values at the lower end of the reference range was more predictive for MetS than a decrease at the upper end of the reference range. Moreover, the confidence intervals for the lower end of the reference range were narrower, implying that the estimation was more precise at lower FT4 levels. According to the mechanism of endocrine regulation, decreased FT4 was accompanied by higher TSH values due to negative feedback. Therefore, we can extend results from the study by Mehran L et al. into the idea that an increase in TSH levels at the higher end of the reference range was more predictive of MetS than an increase at the lower end of the reference range, which is compatible with our findings. However, their results can only be applied to Caucasian patients, and there were only 2,393 participants enrolled in the longitudinal analysis, making their findings relatively less convincing than those of our study, which included 12,463 patients.

**Table 5:** Comparison of different Cohort studies investigating the association between thyroid function and MetS.

Date	Authors	Study type	Race	Age	Initial eligible cases	Follow-up duration	Cases of MetS at follow-up	Variables	Findings
2018	Our studyOur study	Longitudinal	Asian	≥20	12,463	7.2 years (2010–2017)(2010–2017)	480	TSH	HR for MetS by TSH was 1.33 (95% CI = 1.17–1.51; P <0.0001).
2017	Mehran L et al.[16]	Longitudinal	Caucasian	≥20	2,393	9.7 years (2002–2011)(2002–2011)	•2002–2005: 393 •2006–2008: 237 •2009–2011: 320	FT4	ORs for MetS by FT4 was 0.59 (95% CI = 0.39–0.9; *P* was not available).
2016	Kim HJ et al.[14]	Longitudinal	Asian	35–65	13,496	6 years (2006–2012)(2006–2012)	1,664	•FT3 •T3/T4	•OR for MetS in the highest T3 quartile group was 1.249 compared to the lowest T3 quartile group •(95% CI = 1.020–1.529; P = 0.031). •OR for MetS in the highest T3-to-T4 ratio quartile group was 1.458 compared to the lowest T3-to-T4 ratio quartile. (95% CI = 1.141–1.863; P<0.001)
2012	Waring AC et al.[17]	Longitudinal	Caucasian	70–79	2,119	6 years	239	TSH	OR for MetS by TSH was 1.03 (95% CI = 1.01–1.06; *P* = 0.02)
2011	Park SB et al[15].	Longitudinal	Asian	>18	5,998	3 years (2002–2009)(2002–2009)	694	•TSH •FT4	OR for MetS by TSH was 1.103 (*P* = 0.041)

Three other studies, one proposed by Kim et al.[22], one by Warning et al.[17] and one by Park et al.[23] included 13496, 2119 and 5998 eligible participants, respectively, before the beginning of longitudinal follow-up. All three of these studies did not involve a time-to-event investigation of the association between TSH levels and incident MetS because the thyroid-associated values were recorded only at the beginning and at the final follow-up of these studies. Spontaneous normalization of abnormal TSH levels among a significant proportion of patients was reported in many studies [24, 25], resulting in non-differential misclassification and attenuation of the observations. In addition to the above mentioned disadvantages, there were some inconsistencies among the three studies that are discussed below.

In the 2016 study of a Korean population [22], serum T3 levels and the T3-to-T4 ratio were reported to be associated with MetS in middle-aged people; they concluded that both FT4 and TSH had no predictive value for prevalent MetS. Their results were contradictory to ours; there are several possible explanations for this phenomenon. First, alcohol consumption has been reported to have a significant association with prevalent MetS in numerous studies [26–29]. However, alcohol consumption data was not available in their study, implying that their adjustment for covariates might not have been comprehensive enough to accurately evaluate prevalent MetS. Second, the comparison between BMI-defined MetS and WC-defined MetS was controversial in the current studies [30–32]. Nevertheless, they substituted WC with BMI in the definition of MetS, which might be a potential factor interfering with the estimation of prevalent MetS.

In a cohort of community-dwelling subjects aged >70 years from the United States, Warning et al.[17] reported that TSH was associated with prevalent MetS, which was compatible with our cross-sectional analysis. Unfortunately, the number of enrolled patients was much less than that in the present study. In addition, the range of eligible ages in their study was too narrow to apply the results to other populations with different ages. In a 2011 cohort study of a Korean population in 2011 [23], TSH levels were found to be associated with either prevalent or incident MetS. The study population was derived from patients who visited a university hospital for medical evaluations; therefore, it is difficult to apply their results to the general population.

Several current references have proposed possible mechanisms for the association between MetS and TSH levels. The HUNT and Tromsø studies suggested that increases in arterial stiffness and defective vascular relaxation might be reasons for elevated blood pressure associated with increased TSH levels [8, 33, 34]. Higher TSH levels were found to be associated with decreased HDL and increased triglycerides in the HUNT study [9]. Increasing evidence has suggested that thyroid hormone facilitates reverse cholesterol transport in the liver and therefore increases HDL-C activity [35]. Elevated insulin resistance has also been described among patients with subclinical and overt hypothyroidism [36, 37].

Our study has some limitations that should be taken into consideration. First, we did not identify whether or not each of the included participants was taking any medication for thyroid dysfunction. In addition, a lack of FT3 and FT4 measurements had made it impossible to confirm the diagnosis of subclinical hyperthyroidism or hypothyroidism among included patients. Next, homeostatic model assessment for insulin resistance (HOMA-IR), an important predictor for MetS, was also not included in the analysis model because there were no available data concerning HOMA-IR in this study of all Taiwanese. Finally, the race and ethnicity of the participants recruited to the present study were all Asian, restricting the utilization of these findings in other population-based research in the future.

In conclusion, our study highlighted that TSH levels, particularly the highest quartile, were significantly associated with prevalent MetS. In addition, the incidence of MetS was significantly associated with continuous TSH levels, implying that the TSH level was an effective tool for predicting incident MetS. However, further clinical and well-designed longitudinal studies are still needed to determine if decreases of TSH levels might actually be accompanied by a decreased occurrence of MetS.

## References

[1] Alberti KG, Eckel RH, Grundy SM, Zimmet PZ, Cleeman JI, Donato KA, et al Harmonizing the metabolic syndrome: a joint interim statement of the International Diabetes Federation Task Force on Epidemiology and Prevention; National Heart, Lung, and Blood Institute; American Heart Association; World Heart Federation; International Atherosclerosis Society; and International Association for the Study of Obesity. Circulation. 2009;120(16):1640–5. Epub 2009/10/07. doi: 10.1161/CIRCULATIONAHA.109.192644 . 19805654 10.1161/CIRCULATIONAHA.109.192644

[2] Cornier MA, Dabelea D, Hernandez TL, Lindstrom RC, Steig AJ, Stob NR, et al The metabolic syndrome. Endocrine reviews. 2008;29(7):777–822. Epub 2008/10/31. doi: 10.1210/er.2008-0024 ; PubMed Central PMCID: PMCPMC5393149. 18971485 10.1210/er.2008-0024 PMC5393149

[3] Tsou M-T, Chang BC-C, Huang W-H, Hsu C-P. Prevalence of Metabolic Syndrome and Risk Factor Analysis Among Urban Elderly in One Medical Center in Northern Taiwan. International Journal of Gerontology. 8(3):127–32. doi: 10.1016/j.ijge.2013.10.006

[4] Hwang LC, Bai CH, Sun CA, Chen CJ. Prevalence of metabolically healthy obesity and its impacts on incidences of hypertension, diabetes and the metabolic syndrome in Taiwan. Asia Pacific journal of clinical nutrition. 2012;21(2):227–33. Epub 2012/04/18. . 22507609

[5] Nestel P, Lyu R, Low LP, Sheu WH, Nitiyanant W, Saito I, et al Metabolic syndrome: recent prevalence in East and Southeast Asian populations. Asia Pacific journal of clinical nutrition. 2007;16(2):362–7. Epub 2007/05/01. . 17468095

[6] Ruhla S, Weickert MO, Arafat AM, Osterhoff M, Isken F, Spranger J, et al A high normal TSH is associated with the metabolic syndrome. Clinical endocrinology. 2010;72(5):696–701. Epub 2010/05/08. doi: 10.1111/j.1365-2265.2009.03698.x . 20447068 10.1111/j.1365-2265.2009.03698.x

[7] Lai Y, Wang J, Jiang F, Wang B, Chen Y, Li M, et al The relationship between serum thyrotropin and components of metabolic syndrome. Endocrine journal. 2011;58(1):23–30. Epub 2010/12/08. . 21135510 10.1507/endocrj.k10e-272

[8] Asvold BO, Bjoro T, Nilsen TI, Vatten LJ. Association between blood pressure and serum thyroid-stimulating hormone concentration within the reference range: a population-based study. The Journal of clinical endocrinology and metabolism. 2007;92(3):841–5. Epub 2007/01/04. doi: 10.1210/jc.2006-2208 . 17200168 10.1210/jc.2006-2208

[9] Asvold BO, Vatten LJ, Nilsen TI, Bjoro T. The association between TSH within the reference range and serum lipid concentrations in a population-based study. The HUNT Study. European journal of endocrinology. 2007;156(2):181–6. Epub 2007/02/09. doi: 10.1530/eje.1.02333 . 17287407 10.1530/eje.1.02333

[10] Wang F, Tan Y, Wang C, Zhang X, Zhao Y, Song X, et al Thyroid-stimulating hormone levels within the reference range are associated with serum lipid profiles independent of thyroid hormones. The Journal of clinical endocrinology and metabolism. 2012;97(8):2724–31. Epub 2012/06/26. doi: 10.1210/jc.2012-1133 . 22730515 10.1210/jc.2012-1133

[11] Mehran L, Amouzegar A, Tohidi M, Moayedi M, Azizi F. Serum free thyroxine concentration is associated with metabolic syndrome in euthyroid subjects. Thyroid: official journal of the American Thyroid Association. 2014;24(11):1566–74. Epub 2014/07/30. doi: 10.1089/thy.2014.0103 . 25069017 10.1089/thy.2014.0103

[12] Roos A, Bakker SJ, Links TP, Gans RO, Wolffenbuttel BH. Thyroid function is associated with components of the metabolic syndrome in euthyroid subjects. The Journal of clinical endocrinology and metabolism. 2007;92(2):491–6. Epub 2006/11/09. doi: 10.1210/jc.2006-1718 . 17090642 10.1210/jc.2006-1718

[13] Lin SY, Wang YY, Liu PH, Lai WA, Sheu WH. Lower serum free thyroxine levels are associated with metabolic syndrome in a Chinese population. Metabolism: clinical and experimental. 2005;54(11):1524–8. Epub 2005/10/29. doi: 10.1016/j.metabol.2005.05.020 . 16253643 10.1016/j.metabol.2005.05.020

[14] Kim HJ, Bae JC, Park HK, Byun DW, Suh K, Yoo MH, et al Triiodothyronine Levels Are Independently Associated with Metabolic Syndrome in Euthyroid Middle-Aged Subjects. Endocrinology and metabolism (Seoul, Korea). 2016;31(2):311–9. Epub 2016/05/18. doi: 10.3803/EnM.2016.31.2.311 ; PubMed Central PMCID: PMCPMC4923416. 27184017 10.3803/EnM.2016.31.2.311 PMC4923416

[15] Park SB, Choi HC, Joo NS. The relation of thyroid function to components of the metabolic syndrome in Korean men and women. Journal of Korean medical science. 2011;26(4):540–5. Epub 2011/04/07. doi: 10.3346/jkms.2011.26.4.540 ; PubMed Central PMCID: PMCPMC3069574. 21468262 10.3346/jkms.2011.26.4.540 PMC3069574

[16] Mehran L, Amouzegar A, Bakhtiyari M, Mansournia MA, Rahimabad PK, Tohidi M, et al Variations in Serum Free Thyroxine Concentration Within the Reference Range Predicts the Incidence of Metabolic Syndrome in Non-Obese Adults: A Cohort Study. Thyroid: official journal of the American Thyroid Association. 2017;27(7):886–93. Epub 2017/05/10. doi: 10.1089/thy.2016.0557 . 28486021 10.1089/thy.2016.0557

[17] Waring AC, Rodondi N, Harrison S, Kanaya AM, Simonsick EM, Miljkovic I, et al Thyroid function and prevalent and incident metabolic syndrome in older adults: the Health, Ageing and Body Composition Study. Clinical endocrinology. 2012;76(6):911–8. Epub 2011/12/23. doi: 10.1111/j.1365-2265.2011.04328.x ; PubMed Central PMCID: PMCPMC3334430. 22187968 10.1111/j.1365-2265.2011.04328.x PMC3334430

[18] Diagnosis and classification of diabetes mellitus. Diabetes Care. 2014;37 Suppl 1:S81–90. Epub 2013/12/21. doi: 10.2337/dc14-S081 . 24357215 10.2337/dc14-S081

[19] American Diabetes A. Diagnosis and Classification of Diabetes Mellitus. Diabetes Care. 2010;33(Suppl 1):S62–S9. doi: 10.2337/dc10-S062 PMC2797383. 20042775 10.2337/dc10-S062 PMC2797383

[20] Alberti KG, Zimmet P, Shaw J. Metabolic syndrome—a new world-wide definition. A Consensus Statement from the International Diabetes Federation. Diabetic medicine: a journal of the British Diabetic Association. 2006;23(5):469–80. Epub 2006/05/10. doi: 10.1111/j.1464-5491.2006.01858.x . 16681555 10.1111/j.1464-5491.2006.01858.x

[21] Syauqy A, Hsu C-Y, Rau H-H, Chao J. Association of Dietary Patterns with Components of Metabolic Syndrome and Inflammation among Middle-Aged and Older Adults with Metabolic Syndrome in Taiwan. Nutrients. 2018;10(2):143 doi: 10.3390/nu10020143 29382113 10.3390/nu10020143 PMC5852719

[22] Kim HJ, Bae JC, Park HK, Byun DW, Suh K, Yoo MH, et al Triiodothyronine Levels Are Independently Associated with Metabolic Syndrome in Euthyroid Middle-Aged Subjects. Endocrinology and Metabolism. 2016;31(2):311–9. doi: 10.3803/EnM.2016.31.2.311 PMC4923416. 27184017 10.3803/EnM.2016.31.2.311 PMC4923416

[23] Park SB, Choi HC, Joo NS. The Relation of Thyroid Function to Components of the Metabolic Syndrome in Korean Men and Women. Journal of Korean medical science. 2011;26(4):540–5. doi: 10.3346/jkms.2011.26.4.540 PMC3069574. 21468262 10.3346/jkms.2011.26.4.540 PMC3069574

[24] Huber G, Staub JJ, Meier C, Mitrache C, Guglielmetti M, Huber P, et al Prospective study of the spontaneous course of subclinical hypothyroidism: prognostic value of thyrotropin, thyroid reserve, and thyroid antibodies. The Journal of clinical endocrinology and metabolism. 2002;87(7):3221–6. Epub 2002/07/11. doi: 10.1210/jcem.87.7.8678 . 12107228 10.1210/jcem.87.7.8678

[25] Diez JJ, Iglesias P. Spontaneous subclinical hypothyroidism in patients older than 55 years: an analysis of natural course and risk factors for the development of overt thyroid failure. The Journal of clinical endocrinology and metabolism. 2004;89(10):4890–7. Epub 2004/10/09. doi: 10.1210/jc.2003-032061 . 15472181 10.1210/jc.2003-032061

[26] Baik I, Shin C. Prospective study of alcohol consumption and metabolic syndrome. The American journal of clinical nutrition. 2008;87(5):1455–63. Epub 2008/05/13. doi: 10.1093/ajcn/87.5.1455 . 18469271 10.1093/ajcn/87.5.1455

[27] Vieira BA, Luft VC, Schmidt MI, Chambless LE, Chor D, Barreto SM, et al Timing and Type of Alcohol Consumption and the Metabolic Syndrome—ELSA-Brasil. PLoS ONE. 2016;11(9):e0163044 doi: 10.1371/journal.pone.0163044 PMC5028065. 27643787 10.1371/journal.pone.0163044 PMC5028065

[28] Sun K, Ren M, Liu D, Wang C, Yang C, Yan L. Alcohol consumption and risk of metabolic syndrome: a meta-analysis of prospective studies. Clinical nutrition (Edinburgh, Scotland). 2014;33(4):596–602. Epub 2013/12/10. doi: 10.1016/j.clnu.2013.10.003 . 24315622 10.1016/j.clnu.2013.10.003

[29] Djousse L, Arnett DK, Eckfeldt JH, Province MA, Singer MR, Ellison RC. Alcohol consumption and metabolic syndrome: does the type of beverage matter? Obesity research. 2004;12(9):1375–85. Epub 2004/10/16. doi: 10.1038/oby.2004.174 . 15483202 10.1038/oby.2004.174

[30] Aye M, Sazali M. Waist circumference and BMI cut-off points to predict risk factors for metabolic syndrome among outpatients in a district hospital. Singapore medical journal. 2012;53(8):545–50. Epub 2012/09/04. . 22941134

[31] Ryan MC, Fenster Farin HM, Abbasi F, Reaven GM. Comparison of waist circumference versus body mass index in diagnosing metabolic syndrome and identifying apparently healthy subjects at increased risk of cardiovascular disease. The American journal of cardiology. 2008;102(1):40–6. Epub 2008/06/24. doi: 10.1016/j.amjcard.2008.02.096 . 18572033 10.1016/j.amjcard.2008.02.096

[32] Barzin M, Hosseinpanah F, Fekri S, Azizi F. Predictive value of body mass index and waist circumference for metabolic syndrome in 6-12-year-olds. Acta paediatrica (Oslo, Norway: 1992). 2011;100(5):722–7. Epub 2011/01/20. doi: 10.1111/j.1651-2227.2011.02162.x . 21244485 10.1111/j.1651-2227.2011.02162.x

[33] Iqbal A, Figenschau Y, Jorde R. Blood pressure in relation to serum thyrotropin: The Tromso study. Journal of human hypertension. 2006;20(12):932–6. Epub 2006/10/07. doi: 10.1038/sj.jhh.1002091 . 17024137 10.1038/sj.jhh.1002091

[34] Owen PJ, Rajiv C, Vinereanu D, Mathew T, Fraser AG, Lazarus JH. Subclinical hypothyroidism, arterial stiffness, and myocardial reserve. The Journal of clinical endocrinology and metabolism. 2006;91(6):2126–32. Epub 2006/03/16. doi: 10.1210/jc.2005-2108 . 16537677 10.1210/jc.2005-2108

[35] Angelin B, Rudling M. Lipid lowering with thyroid hormone and thyromimetics. Current opinion in lipidology. 2010;21(6):499–506. Epub 2010/10/12. doi: 10.1097/MOL.0b013e3283402e9c . 20935564 10.1097/MOL.0b013e3283402e9c

[36] Maratou E, Hadjidakis DJ, Kollias A, Tsegka K, Peppa M, Alevizaki M, et al Studies of insulin resistance in patients with clinical and subclinical hypothyroidism. European journal of endocrinology. 2009;160(5):785–90. Epub 2009/01/15. doi: 10.1530/EJE-08-0797 . 19141606 10.1530/EJE-08-0797

[37] Dimitriadis G, Mitrou P, Lambadiari V, Boutati E, Maratou E, Panagiotakos DB, et al Insulin action in adipose tissue and muscle in hypothyroidism. The Journal of clinical endocrinology and metabolism. 2006;91(12):4930–7. Epub 2006/09/28. doi: 10.1210/jc.2006-0478 . 17003097 10.1210/jc.2006-0478

